# Lymphatic‑specific magnetic resonance lymphangiography biomarkers for grading lymphedema in animal models

**DOI:** 10.1038/s41598-026-39610-4

**Published:** 2026-02-20

**Authors:** Hwayeong Cheon, Dong-Cheol Woo, Yeon Ji Chae, Ji-wook Kim, Tae-Hyun Shin, Mi-Hyun Kim, Kyung Won Kim, Jae Yong Jeon

**Affiliations:** 1 Rehabilitation Research Center, Biomedical Engineering Research Center, Asan Medical Center, Asan Institute for Life Sciences, Seoul, Republic of Korea; 2 Department of Convergence Medicine, Asan Medical Center, University of Ulsan College of Medicine, Seoul, Republic of Korea; 3 Convergence Medicine Research Center, Asan Medical Center, Asan Institute for Life Sciences, Seoul, Republic of Korea; 4 Inventera Inc., Seoul, Republic of Korea; 5 Research Institute, Trial Informatics Inc., Seoul, Republic of Korea; 6 Department of Radiology and Research Institute of Radiology, Asan Image Metrics, Clinical Trial Center, Asan Medical Center, University of Ulsan College of Medicine, Seoul, Republic of Korea; 7 Department of Rehabilitation Medicine, Asan Medical Center, University of Ulsan College of Medicine, Seoul, Republic of Korea

**Keywords:** Disease severity grading, Imaging biomarker, Lymphedema, Magnetic resonance lymphangiography, Lymph-specific contrast agent, Biomarkers, Diseases, Medical research

## Abstract

Magnetic resonance lymphangiography (MRL) using gadolinium-based contrast agents is valuable for evaluating lymphedema but is limited by its inability to selectively visualize lymphatic structures, hindering accurate diagnosis and severity grading. To overcome this, we demonstrated imaging biomarkers for lymphedema grading using iron oxide-based T1 MRI contrast agent–enhanced MRL (INV-MRL) in animal models. Lymphedema was induced in the hindlimbs of rats by lymph node dissection and radiation, and was successfully established in 29 of 40 animals. Of these, 26 underwent both INV-MRL and near-infrared fluorescence indocyanine green lymphography (NIRF-ICGL) in the same experimental week, and 3 were allocated to excretion studies. Imaging processes were performed under identical injection conditions in the same experimental week. INV-MRL specifically visualized lymphatic vessels, offering superior imaging of deeper lymphatic structures and collateral pathways compared with the predominantly superficial visualization of NIRF-ICGL. Both modalities showed strong concordance in severity grading (Kappa = 0.823), and the Threshold Area Ratio, introduced as a quantitative imaging biomarker, correlated significantly with disease severity. Furthermore, the contrast agent demonstrated rapid excretion within 24 h without notable tissue retention. Collectively, these findings suggest that INV-MRL provides lymphatic-specific qualitative and quantitative biomarkers for lymphedema grading, with the potential to improve diagnostic precision in clinical practice.

**Supplementary Information:**

The online version contains supplementary material available at 10.1038/s41598-026-39610-4.

## Introduction

Lymphedema is the most well-known lymphatic disorder characterized by fluid accumulation due to lymphatic dysfunction, leading to swelling, pain, skin fibrosis, and reduced mobility in the extremities. Lymphedema is a common and representative complication following cancer treatment because it frequently arises after cancer surgeries or radiation therapy for breast, head and neck, or gynecologic cancer^1–6^. The increasing prevalence of lymphedema underscores the importance of precise evaluation following cancer treatment. However, the absence of lymphatic-specific contrast agents and a grading system has limited the utility of radiologic imaging modalities in this context^4,7–10^. Magnetic resonance lymphangiography (MRL) with gadolinium-based contrast agents (GBCAs) is currently the most common imaging technique for visualizing lymphatic vessels (LVs) and lymph nodes (LNs)^11–14^. Although it is a widely used contrast agent, GBCA-based MRL not only visualizes lymphatics but also veins and other tissues, resulting in imaging artifacts, including venous contamination. To overcome this limitation, several studies have explored macromolecular or nanoparticle GBCAs for interstitial MRL, such as the polysiloxane–Gd-DOTA nanoparticle AGuIX, which improves conspicuity of LVs and LNs compared with conventional extracellular GBCAs in rodent hindlimb models^15^. However, these formulations still rely on gadolinium and may exhibit slower clearance and tissue retention, and they have not yet been widely adopted for routine clinical lymphatic imaging. This overlap complicates the accurate assessment of the lymphatic system and hinders imaging biomarkers for establishing reliable grading of lymphedema severity. Additionally, the use of GBCAs for MRL represents an off-label application, further restricting its widespread clinical adoption for lymphedema diagnosis^16–18^.

Conventional iron oxide contrast agents have been used mainly as negative T2 agents, in which the susceptibility-induced dark signal is often indistinguishable from air, hemorrhage, calcification, metal deposition, or thrombus. To address these limitations, INV-001, an iron oxide-based T1 MRI contrast agent, has been developed for lymphatic-specific MRL. This lymphatics-specific MRL aims to enhance the visualization of LVs while minimizing venous contamination and improving the accuracy of lymphedema severity grading. Preclinical studies in healthy animal models, including rats and dogs, have demonstrated that MRL using INV-001 (INV-MRL) predominantly visualizes LVs with no visually apparent venous enhancement under intradermal/subcutaneous injection conditions^18,19^. However, the assessment of lymphatic abnormalities and the discovery of imaging biomarkers to classify severity grading in lymphedema have only been conducted in disease models.

This study aims to find imaging biomarkers for INV-MRL in an animal model of lymphedema and evaluate the medical efficacy for clinical application compared with the images from near-infrared fluorescence indocyanine green lymphangiography (NIRF-ICGL), a reference imaging modality^20,21^.

## Materials and methods

### Generation of hindlimb lymphedema animal models

All experiments were conducted in accordance with the Institutional Animal Care and Use Committee (IACUC) guidelines at the Asan Institute for Life Sciences. The IACUC adheres to the Institute of Laboratory Animal Resources (ILAR) and the Animal Research: Reporting of In Vivo Experiments (ARRIVE) 2.0 guidelines issued by The National Centre for the Replacement, Refinement, and Reduction of Animals in Research (NC3Rs).

**Fig. 1:**
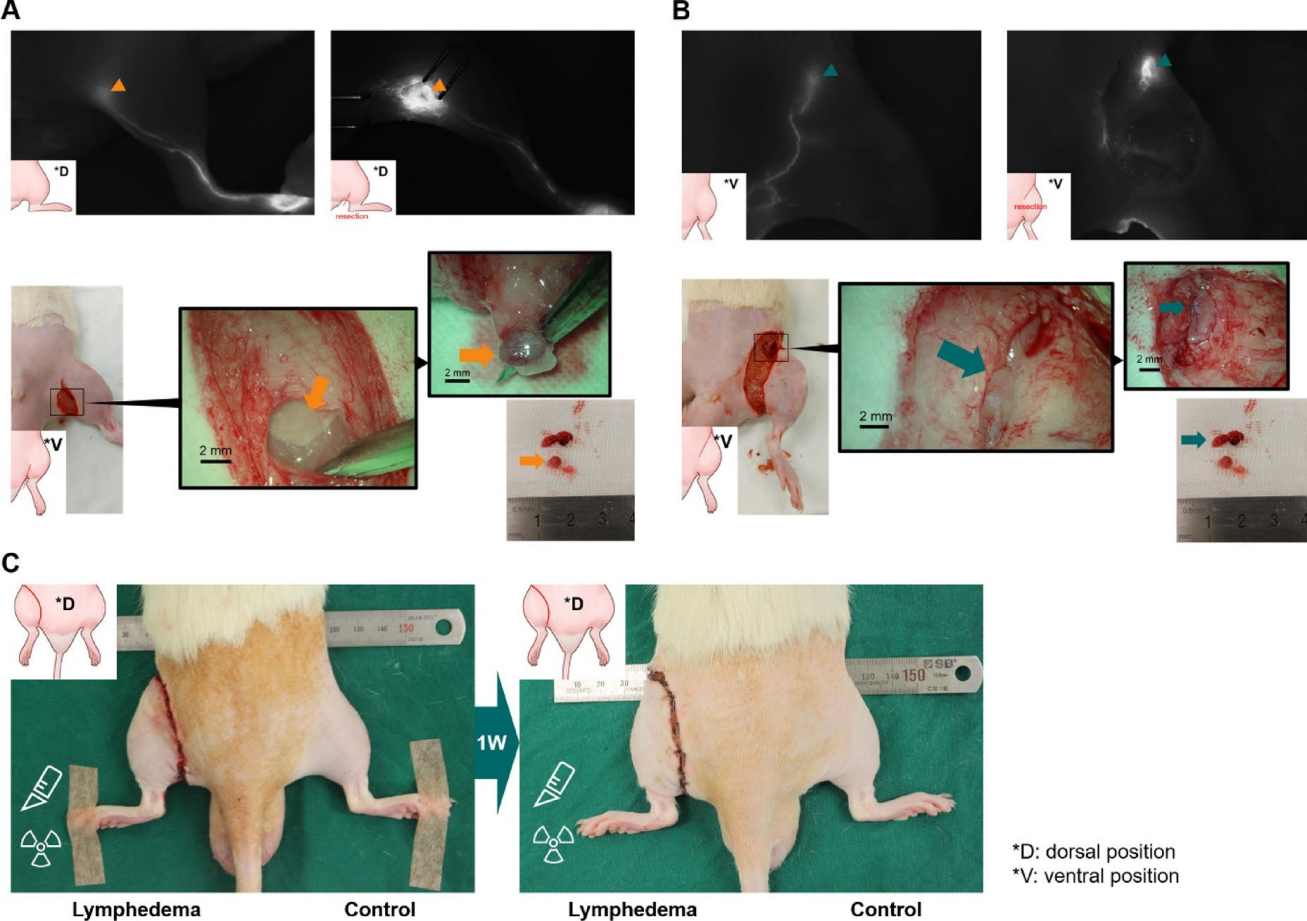
Production of the hindlimb lymphedema model. (**A**) Popliteal lymph nodes (LNs) dissection is performed by making a circumferential incision in the popliteal region, identifying the popliteal LNs, and excising them along with surrounding fat tissue. (**B**) Inguinal LNs dissection involves extending the incision to the inguinal region, identifying the target LNs, and removing them. (**C**) One week after applying radiation to the incision areas, the model is considered successful if ankle swelling is observed.

The experimental protocol/s was/were approved by Asan Medical Center (approval number: 2023-30-167). Forty male Sprague–Dawley rats (8–9 weeks old, 280–320 g) were used. Surgical and radiation protocols adhered to established procedures^22–24^. There is currently no conclusive evidence indicating a sexual difference in the occurrence of lymphedema^25^, and surgical procedures and radiation were used in the animal model in this study to induce physiological lymphatic obstruction. As such, the experimental design did not account for sexual differences. Animals were anesthetized with a combination of tiletamine/zolazepam (Zoletil®; Virbac, Carros, France) at a 50 mg/kg dose and xylazine (Rompun®; Bayer, Leverkusen, Germany) in a 5:1 volume ratio, following initial sedation with 4% isoflurane (Ifran Liq; Hana Pharm Co. LTD., Seoul, Korea) gas mixed with 70% nitrous oxide and 30% oxygen. The hindlimbs were shaved, and a circumferential incision was made in the left groin to expose the inguinal and popliteal LNs^26,27^. LNs and surrounding fat tissue were removed using an electrocautery device (Bovie®; Symmetry Surgical Inc., Antioch, TN) without damaging surrounding tissues. The skin edges were cauterized and sutured to the fascia with 4–0 nylon sutures (Ailee Co. LTD., Pusan, Korea), leaving a 2-mm gap. After surgery, ketoprofen (1 mg/kg, SCD Ketoprofen Inj.; SamChunDang Pharm, Seoul, Korea) was administered intramuscularly (Fig. 1). Two days post-surgery, the surgical site received a focused 20 Gy radiation dose (10 fractions at 1 Gy/min) using an X-ray irradiator (X-Rad 320; Precision X-Ray Inc., Madison, CT). Animals were monitored until full recovery from anesthesia in a warmed environment, and routine clinical assessments (activity, grooming, posture, and food/water intake) were performed thereafter in accordance with IACUC guidelines. Lymphedema was assessed one week later by measuring ankle swelling, defined as a 1 mm increase in ankle diameter in the affected limb compared to the unaffected limb.

### Image acquisition of MRL

We used positive T1 contrast–enhanced MRL in this study because INV-001 predominantly acts as a T1 contrast agent, with the r₁/r₂ ratio of 1.2^18^. MRL scans were performed using a 9.4 T/160-mm preclinical MRI scanner (Agilent, Inc., Santa Clara, CA, USA) with a 72-mm transmit volume coil and surface receiver coil designed for small animals^19^. Animals were anesthetized with 2.0–2.5% isoflurane during the scan. Body temperature was maintained at 37.5 ± 0.5 °C using a warm water bed, and breathing was continuously monitored. MRL was acquired with a coronal 3D time-of-flight (TOF) sequence with saturation bands. Scans parameters were as follows: TR/TE = 10.00/2.54 ms, flip angle = 40°, average = 2, matrix size = 256 × 256 × 192 mm^3^, and FOV = 70 × 70 × 30 mm^3^.

Non-enhanced MRL images were acquired first, followed by a 30-µl intradermal/subcutaneous administration of 15-mM INV-001 (Inventera Inc., Seoul, Republic of Korea) solution into the web space between the 3rd and 4th toes of both control and affected limbs using a 26-gauge insulin syringe (BD Medical-Diabeters Care, Franklin Lakes, NJ) for contrast-enhanced MRL. MRL with INV-001 (INV-MRL) was performed every 16 min for 96 min. Coronal 3D TOF images were generated by acquiring stacks of x-y, y-z, and z-x slices. Images were reconstructed as coronal, sagittal, and 3D maximum-intensity projection reconstructions to visualize the LVs clearly (Supplementary Fig. 1 and Supplementary Video 1). Venous contamination on INV-MRL was qualitatively assessed by two readers using non-enhanced images as a reference and considering the anatomical course, branching pattern, and temporal enhancement of each enhancing structure, as well as correlation with NIRF-ICGL findings.

### Image acquisition of NIRF-ICGL

NIRF-ICGL images were acquired within three days after INV-MRL scans using a custom imaging system with 4.2-watt power LEDs (730 nm peak, LST1-01G01-FRD1-00; Opulent Americas, Raleigh, NC) and a 2-inch bandpass filter (FF01-832/27-50-D; Semrock, West Henrietta, NY) for fluorescence detection^20^. A 20-µL solution of ICG fluorescent dye (Diagno Green inj.; Daiichi Sankyo, Tokyo, Japan) in bovine serum albumin (2.5 mg/ml; Sigma-Aldrich, Saint Louis, MS) was injected into the paw in the same manner as the INV-001 injection. Images were captured 30 min after ICG injection. Owing to NIRF-ICGL’s penetration depth limitations, images were acquired separately from the dorsal (D, dorsal NIRF-ICGL) and ventral (V, ventral NIRF-ICGL) positions (Supplementary Fig. 2).

### Pain management during contrast administration

To minimize discomfort and motion during contrast administration for imaging, injections were performed under brief inhalational anesthesia with isoflurane. Injections were delivered using 26-gauge insulin syringes with minimal volumes (20 µl) appropriate to each modality and strict aseptic technique. No local anesthetic was infiltrated at the injection sites and no additional systemic analgesics were administered immediately before imaging to avoid potential effects on lymphatic flow or tracer kinetics. Animals were continuously observed during imaging and recovered on a warming surface until fully ambulatory.

### Endpoint for animal model formation and imaging process

The primary endpoint was successful unilateral hindlimb lymphedema induction 1-week post-procedure and radiation, predefined as an ankle diameter increase ≥ 1 mm in the affected limb versus the contralateral limb. Secondary endpoints included survival and recovery to the imaging time point without IACUC-triggering complications. Exclusion/humane endpoints comprised failure to meet the ≥ 1 mm swelling criterion or predefined welfare concerns (for example, marked weight loss, persistent pain/distress, or wound complications), prompting withdrawal and humane euthanasia per IACUC policy. For imaging, the endpoint was clear delineation of LVs or lymphatic drainage on images after contrast administration under stable physiologic monitoring (37.5 ± 0.5 °C, continuous respiration) with image quality suitable for grading/quantification and cross-modality comparison.

### Grading classification based on qualitative imaging biomarkers of NIRF-ICGL and INV-MRL

Two experienced readers (H.C. and J.Y.J., both with over 10 years of experience in animal lymphedema research) analyzed NIRF-ICGL images to identify imaging biomarkers and graded the severity of lymphedema. Two other readers (D.C.W. and K.W.K., both with over 10 years of animal MRI research experience) analyzed INV-MRL images, focusing on the LV imaging biomarkers, and graded the lymphedema severity.

All four readers jointly found imaging biomarkers and developed the grading systems for NIRF-ICGL and INV-MRL. NIRF-ICGL Grading: Based on the dermal backflow imaging biomarkers in clinical practice^28^, the grading system was as follows (Supplementary Fig. 3):

- *Grade 0*: (normal condition): Collecting LVs are visible in a linear pattern without dermal backflow.
- *Grade 1*: (minimally impaired drainage): A splash pattern, reflecting collateral LVs bypassing the affected area.
- *Grade 2*: (mildly impaired drainage): A stardust pattern, indicating scattered lymphatic fluid leakage around the distal region, with a splash pattern extending throughout the limb.
- *Grade 3*: (moderately impaired drainage): The stardust pattern extends proximally.
- *Grade 4*: (severely impaired drainage): A diffuse pattern, showing accumulated lymphatic fluid in surrounding tissues.
- *Grade 5*: (complete obstruction): A blackout pattern, with no ICG visible beyond the injection site due to a lack of fluid movement.

The INV-MRL grading system was based on the visibility of dilated LVs, collateral pathways, and fluid diffusion into surrounding tissues, aligning with NIRF-ICGL and prior studies^11,29–33^. Grades were as follows (Supplementary Fig. 3):

- *Grade 0*: Collecting LVs are visible from the injection site to the LNs.
- *Grade 1*: Collateral pathways extend from LVs into surrounding tissue in a pattern, resembling the stardust pattern in NIRF-ICGL.
- *Grade 2*: Collateral pathways extend from the existing collecting LVs to the surrounding tissue in a scattered pattern, resembling the stardust pattern in NIRF-ICGL.
- *Grade 3*: Collateral pathways expand, and LVs are dilated compared to the control limb.
- *Grade 4*: Diffuse areas of contrast enhancement in the surrounding reflect pooled contrast agent in the interstitial fluid.
- *Grade 5*: No contrast agent is visible beyond the injection site, indicating a complete lack of lymphatic drainage.

### Quantitative analysis of imaging biomarkers in lymphedema limbs

We performed quantitative analysis of these imaging biomarkers by defining the threshold area ratio (TAR) as a parameter to determine the severity of lymphedema in both NIRF-ICGL and INV-MRL. The TAR was calculated by dividing the enhanced threshold area (brightness above the median value of LVs by contrast enhancement) by the total region of interest (ROI) area (Eq. 1).

1 $$TAR\;(\mathrm{a}.\mathrm{u}.)=\frac{Enhanced\;threshold\;area}{Total\;ROI\;area}$$

TAR values were obtained using ImageJ open-source software (version 1.53c; http://rsbweb.nih.gov/ij/; NIH, Bethesda, MD). A consistent ROI, from the ankle to the hip joint, was established. The threshold area for LVs was determined in control limbs and applied to lymphedema limbs. The threshold range was set at stage 0, where INV-MRL and dorsal NIRF-ICGL images selected only the LVs without background, and this established range was used to analyze all images (Supplementary Fig. 4).

### Statistical analysis

Paired t-tests were used to evaluate differences in ankle diameter between the lymphedema and control limb, both before and after the model formation. Inter-rater agreement on lymphedema severity between NIRF-ICGL and INV-MRL was assessed using the weighted Cohen’s Kappa coefficient, with interpretation as follows: 0.00–0.20 (none to slight), 0.21–0.40 (fair), 0.41–0.60 (moderate), 0.61–0.80 (substantial), and 0.81–1.00 (excellent) agreement. Pearson’s correlation coefficient was calculated to assess the relationship between the TAR values from INV-MRL and dorsal/ventral NIRF-ICGL and lymphedema severity grades. Statistical analyses were conducted using GraphPad Prism 10 (version 10.1.2; GraphPad Software, Inc., Boston, MA).

## Results

### Lymphedema model

Lymphedema was successfully induced in 29 of the 40 rats subjected to the surgical and radiation procedure, as evidenced by ankle swelling. The ankle diameter of the lymphedema-affected limbs was significantly larger compared to the control limbs (mean difference = 1.4 mm, *P* < 0.001, paired t-test), as shown in Supplementary Fig. 5. Among these 29 rats, 3 were allocated to a pilot study to optimize imaging acquisition methods and assess the excretion of INV-001. The remaining 26 rats underwent image analysis and grading of lymphedema severity using INV-MRL and NIRF-ICGL.

### Image features of NIRF-ICGL and INV-MRL to find imaging biomarkers

In all 26 rats, LVs were visualized in both control and lymphedema limbs on NIRF-ICGL and INV-MRL, as confirmed by two independent readers for each imaging modality. No macroscopic venous enhancement suggestive of venous contamination was observed on INV-MRL in any animal models.

**Fig. 2:**
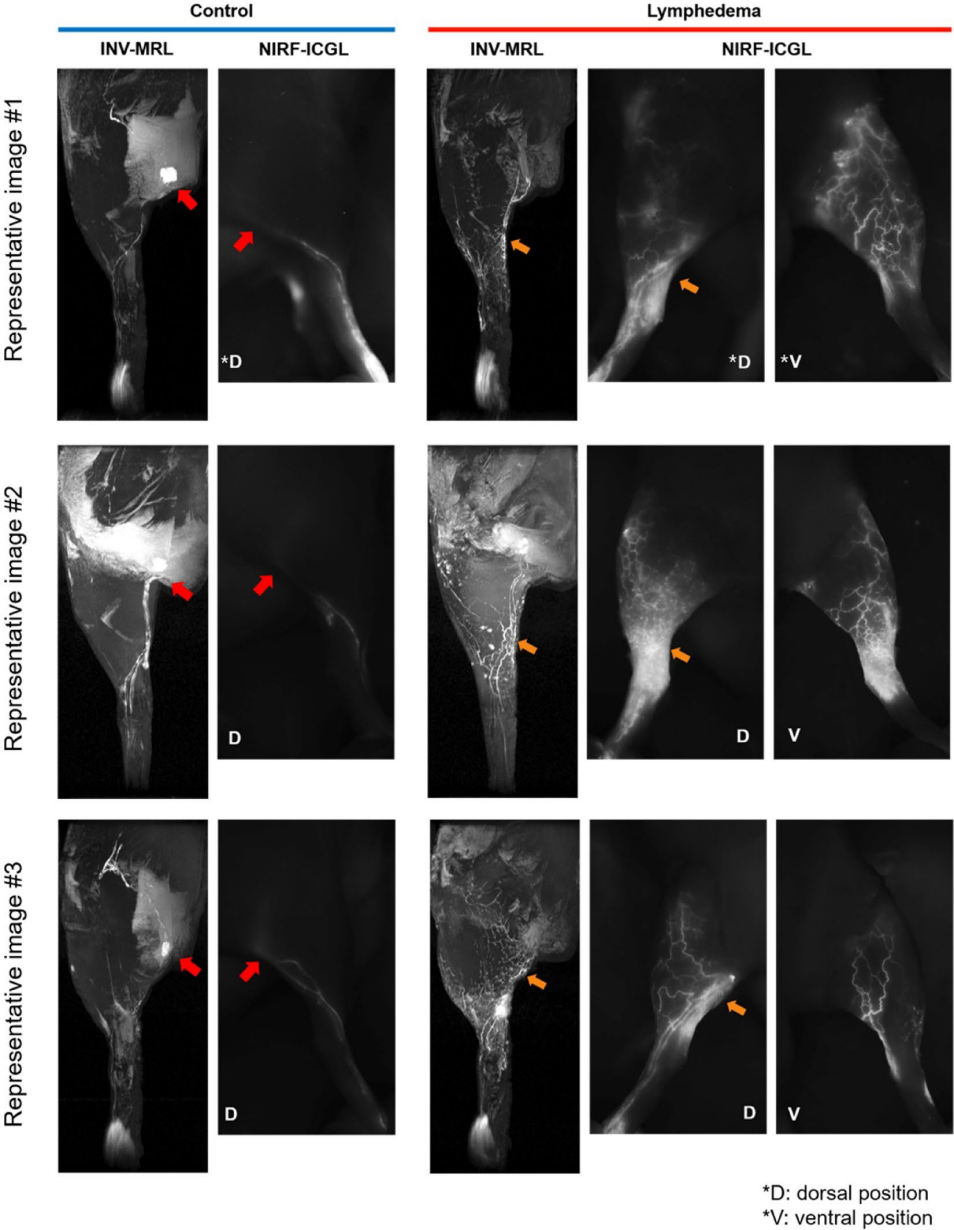
Representative images of INV-MRL and NIRF-ICGL in dorsal (D) and ventral (V) positions. In control limbs, popliteal LNs were visible in both modalities (red arrow). Orange arrows indicate areas with a diffuse pattern in NIRF-ICGL, where the contrast agent spreads broadly over the skin surface, obscuring the underlying lymphatic vessels (LVs). However, these hidden LVs are visible in INV-MRL.

In the control hindlimbs, both NIRF-ICGL and INV-MRL identified collecting LVs and popliteal LNs. INV-MRL displayed almost identical LV morphology to NIRF-ICGL in the dorsal view, though the collecting LVs were more distinct on INV-MRL (Fig. 2).

In the lymphedema-affected hindlimbs, the imaging features differed between modalities. NIRF-ICGL predominantly visualized superficial LVs, which appeared as fine networks. In contrast, INV-MRL depicted deeper LVs, which were relatively larger. Dermal backflow, indicative of lymphatic fluid leakage into surrounding tissues, was more prominent in NIRF-ICGL compared to INV-MRL. Collateral LVs were clearly visualized with INV-MRL but were challenging to observe using NIRF-ICGL due to the obscuration caused by the spread of ICG on the skin surface (Fig. 2).

### Grades of lymphedema severity in NIRF-ICGL and INV-MRL based on imaging biomarkers

**Fig. 3:**
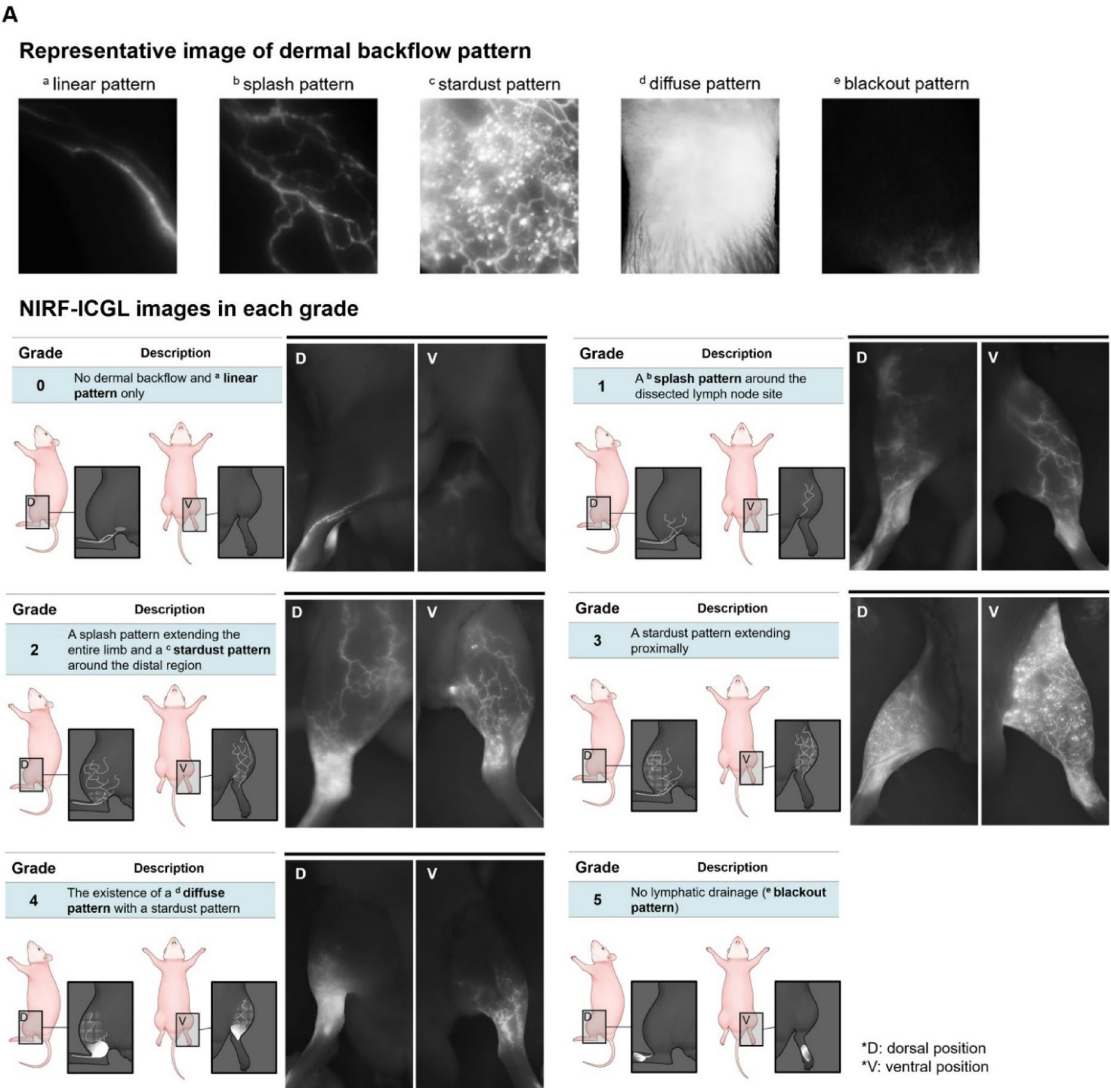

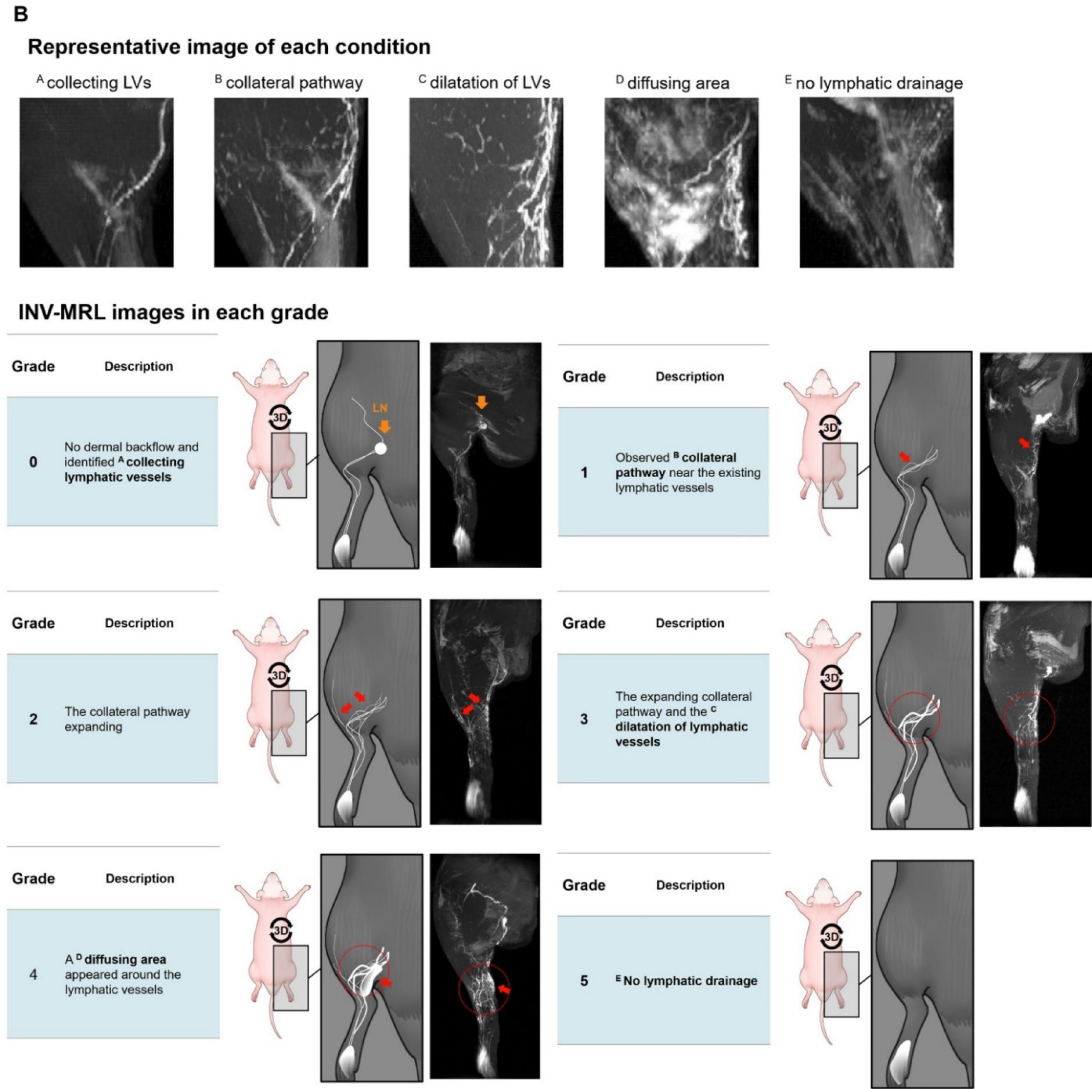
Representative patterns for imaging biomarkers and images of (**A**) NIRF-ICGL and (**B**) INV-MRL for each grade. (Note: No grade 5 animal models were included in this study.)

Both NIRF-ICGL and INV-MRL demonstrated a transition from linear lymphangiographic patterns (visualizing only LVs) to diffuse patterns (visualizing leaked lymphatic fluid in surrounding tissue) as lymphedema severity increased. NIRF-ICGL clearly identified dermal backflow patterns, including splash, stardust, and diffuse features, but poorly visualized collateral LVs (Fig. 3A). Conversely, INV-MRL provided a clearer visualization of collateral LVs but depicted dermal backflow features less prominently, with these features becoming evident at higher grades (Fig. 3B).

Using the NIRF-ICGL grading system, 5 rats were classified as grade 1, 6 as grade 2, 7 as grade 3, and 8 as grade 4. INV-MRL classified 6 rats as grade 1, 5 as grade 2, 9 as grade 3, and 6 as grade 4. No grade 5 lymphedema was observed. The Kappa coefficient was 0.823 (95% confidence interval; 0.676‒0.971), indicating excellent agreement between NIRF-ICGL and INV-MRL.

### TAR biomarker for lymphedema severity quantification

**Fig. 4:**
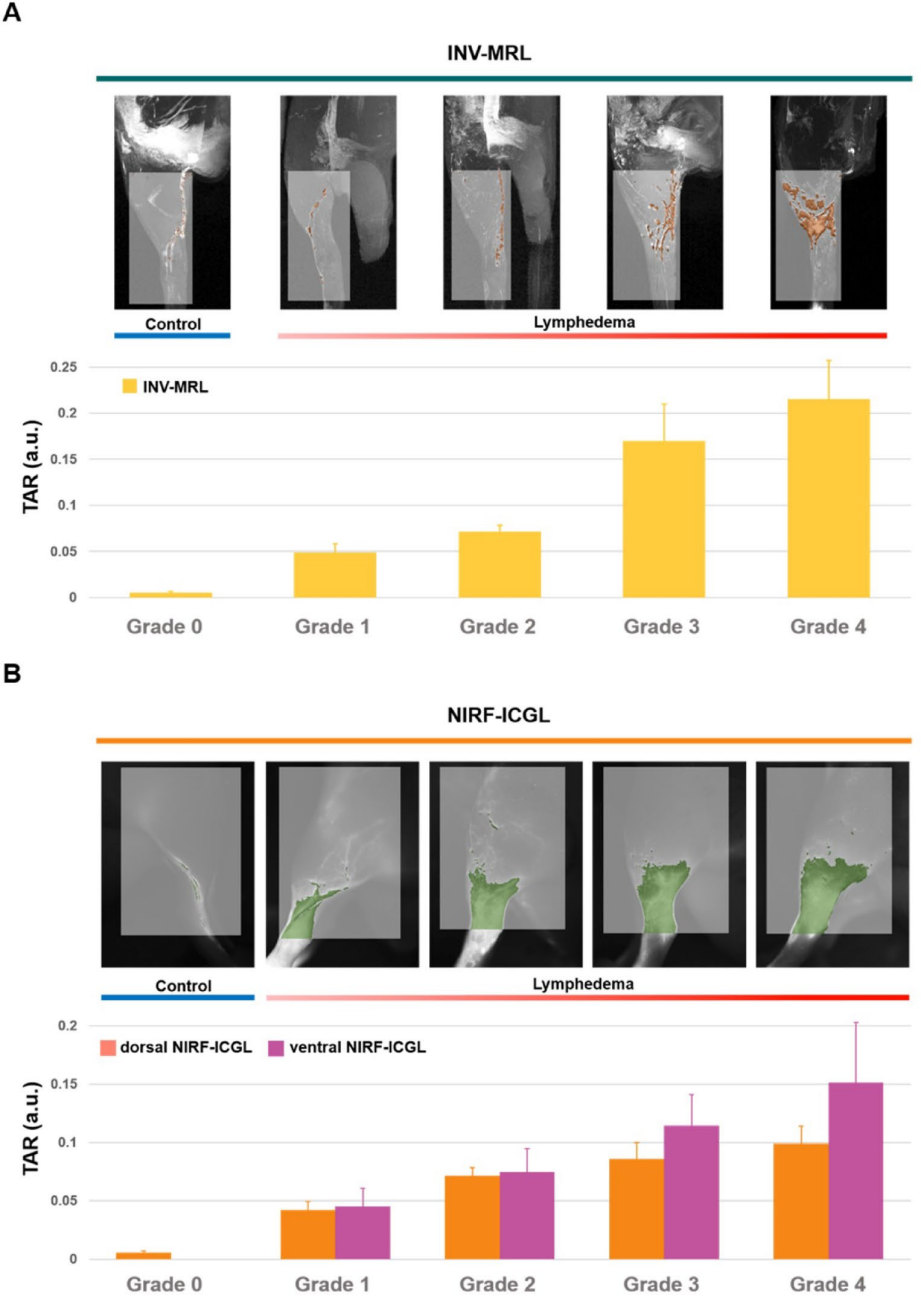
Threshold area ratio (TAR) values in control and lymphedema-affected limbs in (**A**) INV-MRL and (**B**) dorsal and ventral NIRF-ICGL. Average TAR values increase progressively with advancing lymphedema severity from grade 0 (control) to higher grades.

The TAR values increased with higher lymphedema severity grades for both INV-MRL and NIRF-ICGL (Fig. 4, Supplementary Fig. 6). The Pearson correlation coefficients between TAR and lymphedema severity grades were 0.769 for INV-MRL, 0.729 for dorsal NIRF-ICGL, and 0.602 for ventral NIRF-ICGL (Supplementary Fig. 7). The TAR values for INV-MRL showed significant correlation with TAR values for NIRF-ICGL, with coefficients of 0.51 (*P* < 0.05) for dorsal NIRF-ICGL and 0.42 (*P* < 0.05) for ventral NIRF-ICGL.

### Excretion study

In three rats, the excretion study of INV-001 was evaluated 24 hours after administration to determine its retention in the body. INV-001 was not detected at the injection site or in lymphatic vessels at 24 hours in both control and lymphedema limbs. T1 and T2 relaxation times in the liver and kidney, measured via T1 and T2 mapping, showed no significant changes at 24 hours compared to baseline (0 hour) (Supplementary Fig. 8). These findings suggest that INV-001 was effectively excreted from the body within 24 hours in both control and lymphedema-affected limbs.

## Discussion

In this preclinical study, INV-MRL provided clear visualization of LVs in lymphedema-affected limbs with no visually apparent venous contamination on the acquired images, and the grading of lymphedema severity demonstrated excellent agreement with NIRF-ICGL. This represents a marked improvement over conventional MRL using GBCAs, where venous contamination is a common issue, complicating diagnosis^34–36^. Compared with previously described macromolecular GBCAs for interstitial MRL^15^, INV-001 offers several conceptual advantages as a lymphatic-specific contrast agent. Because INV-001 is iron-oxide–based, it circumvents gadolinium-related concerns such as tissue deposition and nephrogenic systemic fibrosis, which is particularly relevant for repeated lymphatic imaging. In our studies, INV-MRL provided lymphatic-dominant enhancement with minimal venous contamination and no evident residual enhancement at the injection site or major organs on delayed images. It suggests efficient clearance under the present dosing and timing conditions, and can be evidence that increases the possibility of clinical use. In a clinical setting, INV-MRL combined with anatomical imaging such as T1-weighted/T2-weighted imaging could provide robust diagnostic information for developing effective treatment strategies for lymphedema patients by visualizing the network of the lymphatic system and body components.

The imaging biomarker derived from INV-MRL presents several notable characteristics, establishing its utility as a lymphatic-specific digital biomarker. Firstly, these findings indicate that INV-MRL provides complementary qualitative and quantitative imaging biomarkers of lymphedema severity. Qualitatively, INV-MRL depicts characteristic lymphatic abnormalities, including LV dilation, collateral pathways, and dermal backflow. Quantitatively, the TAR offers an area-based measure of contrast enhancement that correlates with imaging-based severity grades and limb swelling, supporting its potential use as an objective biomarker for lymphedema assessment. Secondly, the defined molecular size of INV-001 (3.6 nm) significantly reduces nonspecific tissue diffusion, enhancing biomarker specificity. This characteristic enables precise quantification of abnormal lymphatic drainage patterns. Additionally, the relatively large hydrodynamic diameter of INV-001 facilitates selective uptake by LVs upon intradermal or subcutaneous injection, resulting in predominant visualization of LVs with minimal venous interference and enabling accurate digital tracking of lymphatic system alterations associated with disease onset and progression^18,19^.

The characteristics of imaging biomarkers provided by INV-MRL distinguish it significantly from NIRF-ICGL. Specifically, INV-MRL demonstrates superior capabilities as a digital biomarker due to its superior depth penetration, making it particularly effective for delineating LVs and collateral pathways in lymphedema conditions, compared to the superficial imaging characteristics of NIRF-ICGL^21,37,38^. Moreover, the manifestation of dermal backflow in INV-MRL predominantly occurs in more advanced stages of lymphedema severity, whereas NIRF-ICGL detects diverse patterns of dermal backflow even in the early stages of the disease. Consequently, INV-MRL provides clearer and more detailed quantitative information regarding pathological lymphatic anatomy in severe lymphedema cases, emphasizing its value as an advanced imaging biomarker for precise disease assessment.

Currently, INV-MRL is an important imaging modality for evaluating lymphatic drainage, assessing lymphedema severity, and guiding treatment strategies, such as lymphovenous anastomosis or vascularized lymph node transfer^20,39–41^. Recently, studies have classified lymphatic drainage patterns based on dermal backflow staging in NIRF-ICGL images to distinguish lymphedema severity^42–44^. To facilitate the clinical translation of INV-MRL, we developed qualitative lymphedema severity grading criteria based on established criteria using dermal backflow biomarkers in NIRF-ICGL. Our newly developed grading system demonstrated strong agreement (Kappa = 0.823) with NIRF-ICGL assessments, reinforcing the reliability of INV-MRL as a robust digital biomarker capable of capturing detailed pathological features of the lymphatic system.

In both NIRF-ICGL and INV-MRL, lymphangiographic imaging biomarkers shift from linear to diffuse forms with increased lymphedema severity. Under normal physiological conditions, interstitial fluid in the extracellular matrix is collected and transferred into the lymphatic system. Consequently, the ICG agent in the interstitial fluid is absorbed by LVs, appearing as a linear pattern in NIRF-ICGL imaging under normal physiological conditions. When lymphatic obstruction leads to lymphedema, drainage into collecting LVs is impaired. The lymphatic system compensates by forming collateral pathways, which appear as a “splash” pattern. As lymphatic obstruction worsens, leakage occurs from both collecting and collateral LVs, resulting in a “stardust” pattern characterized by scattered points. In advanced stages, interstitial fluid accumulates in the extracellular matrix, creating a diffuse pattern. In this case, the contrast agent spread across the skin surface makes it difficult to identify LVs under the skin using NIRF-ICGL. However, INV-MRL reduces imaging obscuration and enables visualization of the internal existing LVs below the skin. In the most severe cases of chronic lymphedema, fluid movement ceases, and contrast is no longer visible beyond the injection site, a phenomenon termed the “blackout pattern”^28^, and it was not observed in the current study because our experiments involved imaging within a relatively short timeframe following the onset of lymphatic impairment.

Additionally, we introduced the TAR as a quantitative imaging biomarker that accurately reflects disease severity. TAR quantitatively measures the extent of contrast spread, correlating directly with progressive lymphatic fluid leakage and disease severity. TAR values increased proportionally with lymphedema severity, suggesting TAR may serve as a reliable quantitative parameter that aligns with qualitative criteria. Our findings revealed TAR values obtained from INV-MRL closely correlated with dorsal NIRF-ICGL findings, highlighting the enhanced capability of INV-MRL as a digital biomarker for the early and precise detection of lymphatic abnormalities. Through these quantitative imaging biomarkers and multimodal comparison with NIRF-ICGL, INV-MRL may provide a framework for objective, lymphatic-specific grading of lymphedema severity that has not yet been systematically established for existing contrast agents.

In summary, the lymphatic-specific INV-MRL accurately reflected lymphatic structures with minimal venous interference, provided quantitative and qualitative assessments of disease severity, and complemented NIRF-ICGL imaging. Its advanced imaging biomarker characteristics for grading lymphedema, including visualization of deeper lymphatic structures and minimized imaging interference, underscore its potential to overcome existing limitations in conventional lymphatic imaging modalities. However, detailed head-to-head comparisons of signal-to-noise ratio and cost-effectiveness between INV-001 and other formulations were beyond the scope of this study. In addition, we did not perform dedicated MR venography or serial blood sampling, and therefore, subtle venous intravasation of INV-001 cannot be completely excluded, particularly in more advanced or heterogeneous states of lymphatic dysfunction.

## Supplementary Information

Below is the link to the electronic supplementary material.

Supplementary Material 1

Supplementary Material 2

## Data Availability

All photographs, imaging data, and videos included in this manuscript were generated by the authors Hwayeong Cheon and Dong-Cheol Woo. All data generated or analysed during this study are included in this published article and its supplementary information files.

## Abbreviations

MRL: Magnetic resonance lymphangiography (lymphography)
GBCAs: Gadolinium-based contrast agents
LVs: Lymphatic vessels
LNs: Lymph nodes
NIRF-ICGL: Near-infrared fluorescence indocyanine green lymphangiography
TAR: Threshold area ratio
ROI: Region of interest
INV-MRL: Magnetic resonance lymphangiography (lymphography) using INV-001
T1/T2: Longitudinal (T1) and transverse (T2) relaxation times in MRI
