# Supplementary material for: Lymphatic‑specific magnetic resonance lymphangiography biomarkers for grading lymphedema in animal models
Source: Sci Rep. 2026 Feb 20;16:10008. doi: 10.1038/s41598-026-39610-4 (PMC13021956; doi:10.1038/s41598-026-39610-4)

Supplementary Figure 1. Representative 3D maximum-intensity projection reconstruction images of INV-MRL (See also Supplementary Video 1).

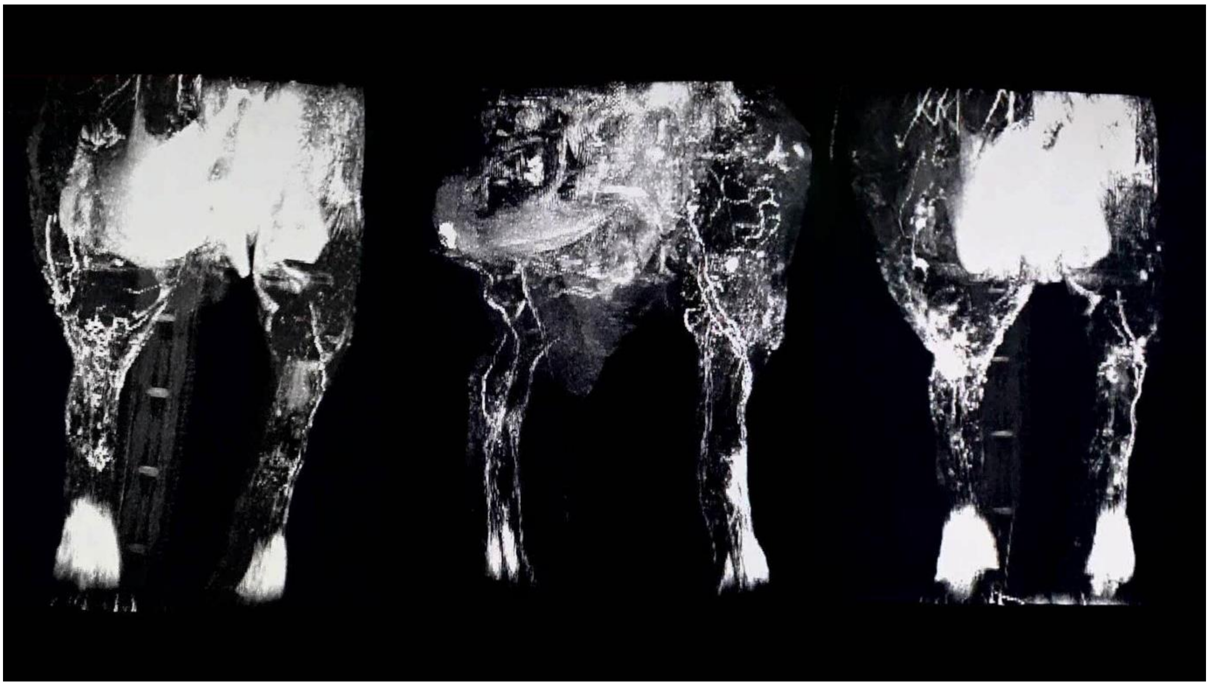

Supplementary Figure 2. Near-infrared indocyanine green lymphangiography (NIRF-ICGL) process. (**A)** The customized NIRF-ICGL system for this study and its excitation and emission spectrum. (**B)** Animal posture and ROI location to obtain images. (**C)** NIRF-ICGL of control and lymphedema limbs

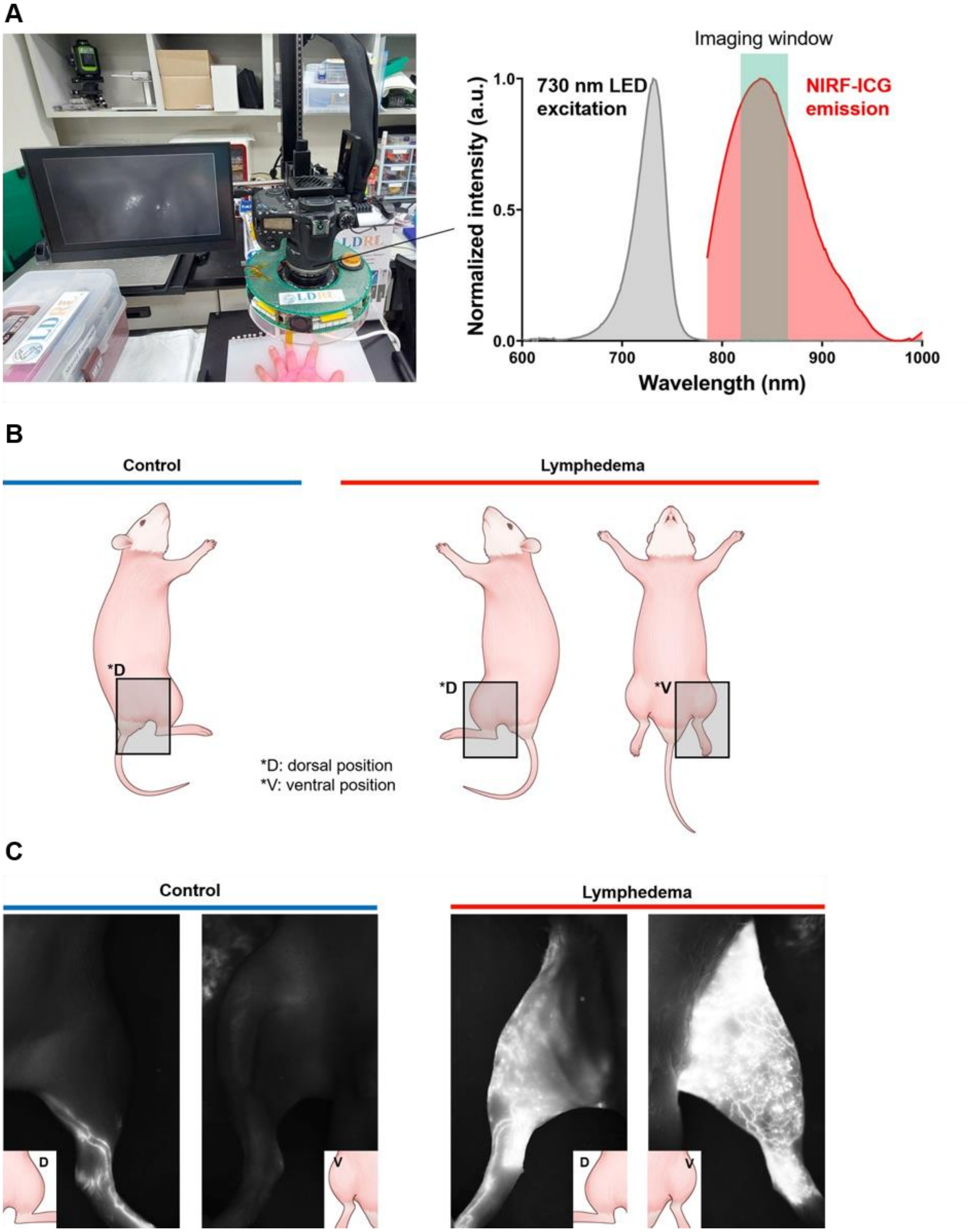

Supplementary Figure 3. Qualitative lymphedema severity grading criteria for **(A)** near-infrared fluorescence indocyanine green lymphography (NIRF-ICGL) and **(B)** intranodal magnetic resonance lymphangiography (INV-MRL).

A

NRF-ICGL

Grade	Description	Diagram
0	No dermal backflow and linear pattern only	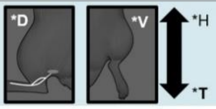
1	A splash pattern around the dissected lymph node sites	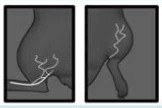
2	A splash pattern extending the entire limb and a stardust pattern around the distal region	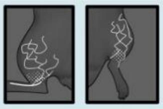
3	A stardust pattern extending proximally	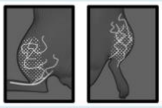
4	The existence of a diffuse pattern with a stardust pattern	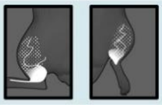
5	No lymphatic drainage (blackout pattern)	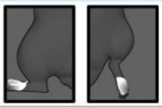

*D: dorsal position
*V: ventral position
*H: head direction
*T: tail direction

B

INV-MRL

Grade	Description	Diagram
0	No dermal backflow and identified collecting LVs (and LNs)	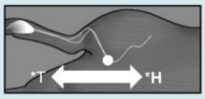
1	Observed collateral pathway near the existing LVs	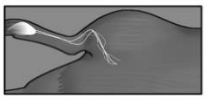
2	The collateral pathway expanding	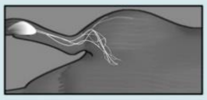
3	The expanding collateral pathway and the dilatation of LVs	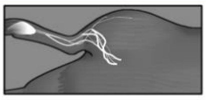
4	A diffusing area appeared around the LVs	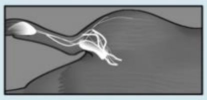
5	No lymphatic drainage	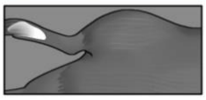

*H: head direction
*T: tail direction

Supplementary Figure 4. Definition of threshold area ratio (TAR) and illustrations of quantitative evaluation for the lymphedema limbs.

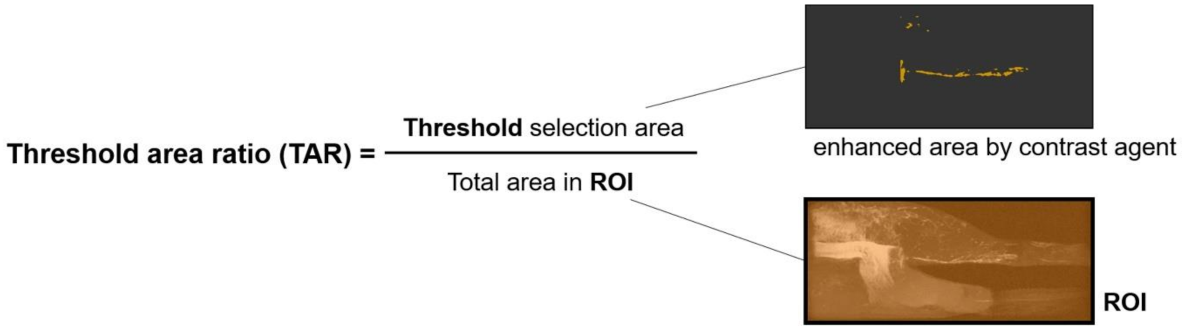

Supplementary Figure 5. The significant changes in ankle diameter of control and lymphedema limbs before and after surgery and radiation. The inset shows a photo of a swollen ankle caused by lymphedema. **** means P<0.0001.

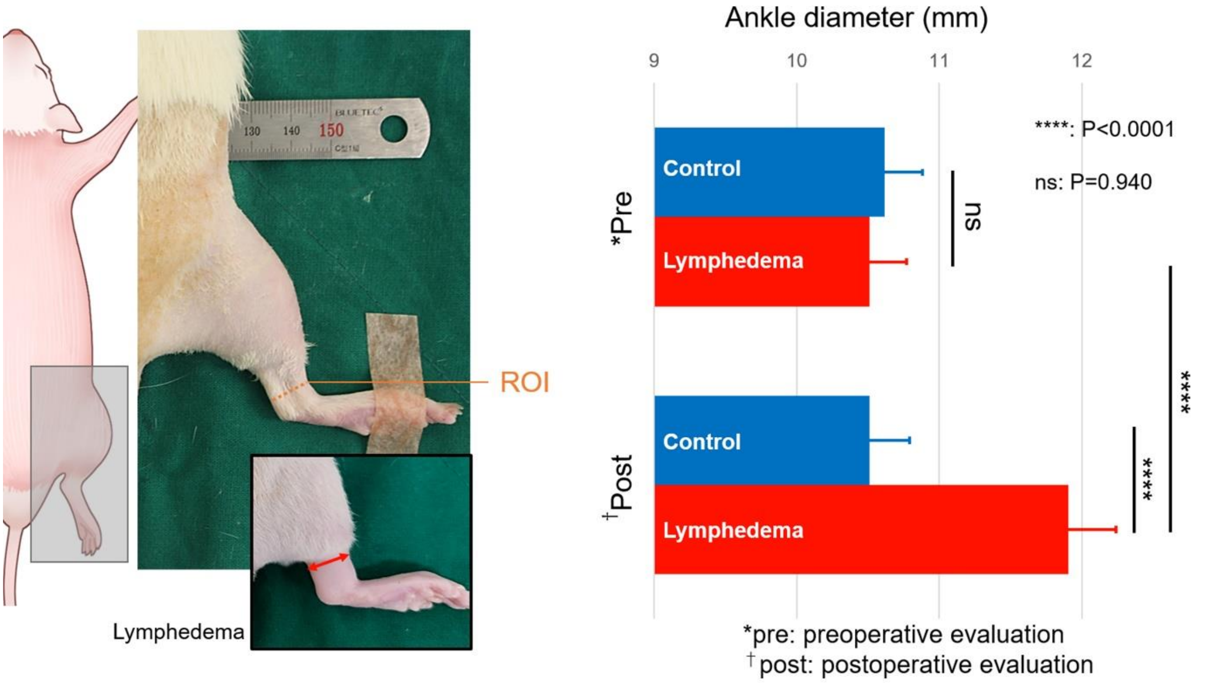

Supplementary Figure 6. In dermal backflow staging for NIRF-ICGL, the patterns that simplify them, and the change of the threshold area ratio (TAR) according to them. Because the area of white enhancement (caused by ICG fluorescence) was proportional to the stage of lymphedema, the value of TAR also increased.

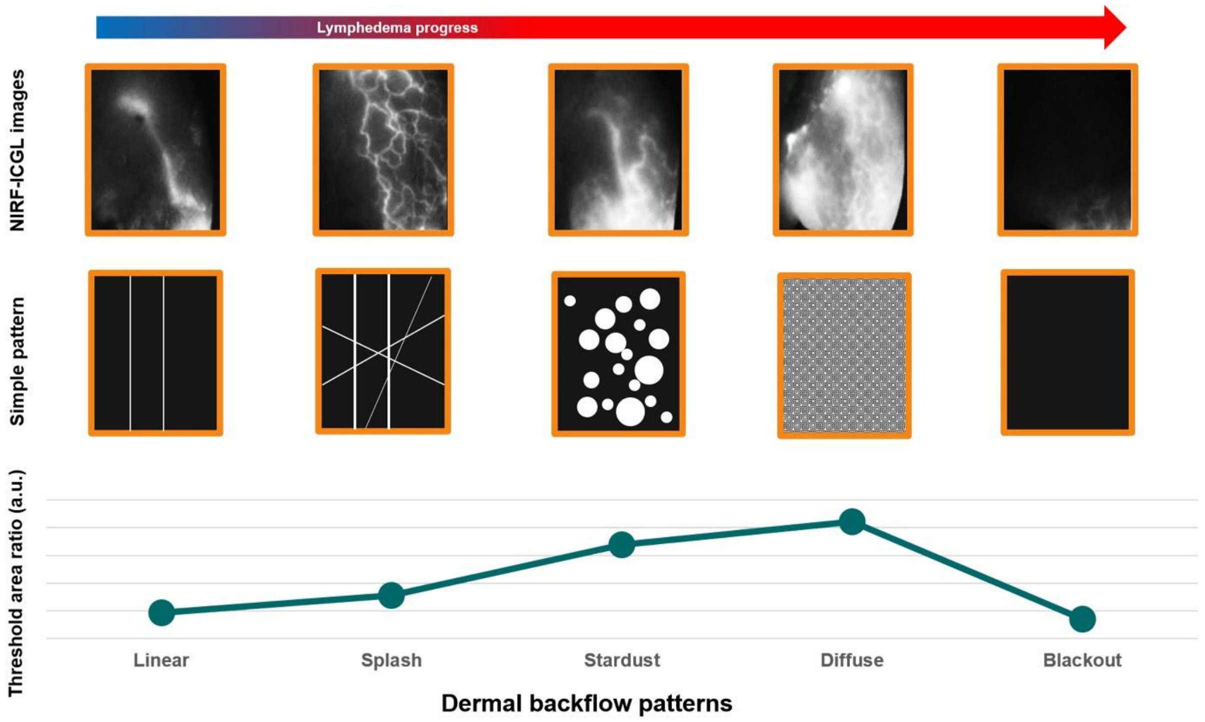

Supplementary Figure 7. Correlation between TAR in each imaging modality and lymphedema severity grades. The Pearson's correlation coefficient between the TRL and lymphedema severity grade was 0.769 for INV-MRL (P <0.001), 0.729 for dorsal NIRF-ICGL (P <0.001), and 0.602 for ventral NIRF-ICGL (P <0.05).

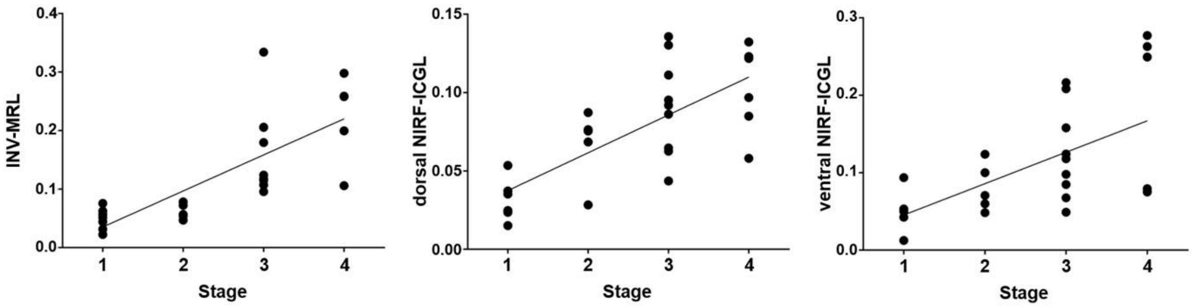

Supplementary Figure 8. Extraction of INV-001 in the liver (L), medulla (M), and cortex (C).

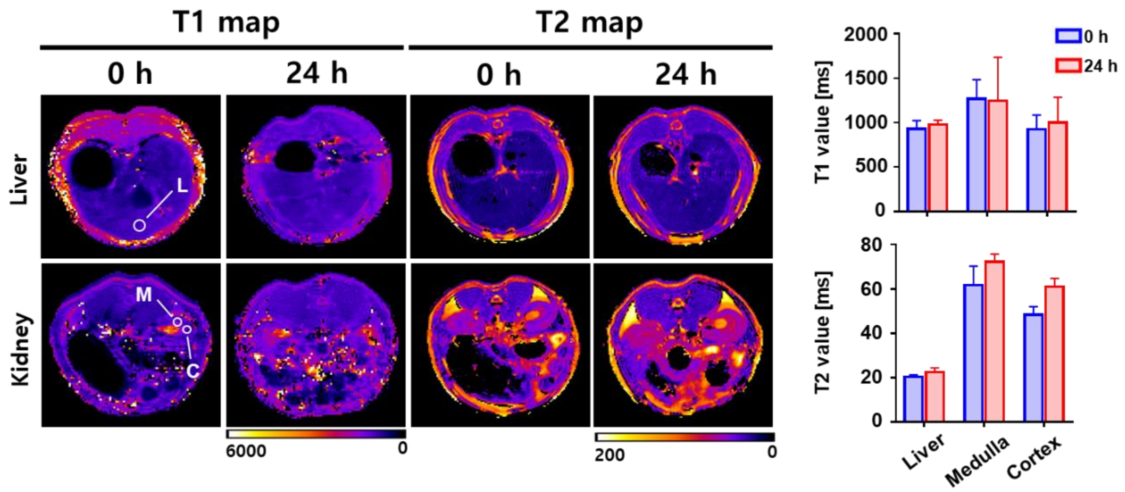

Supplement: Supplementary file 2 — Supplementary Material 2 [file 41598_2026_39610_MOESM2_ESM.pdf]
